# Transient and Persistent Pain Induced Connectivity Alterations in Pediatric Complex Regional Pain Syndrome

**DOI:** 10.1371/journal.pone.0057205

**Published:** 2013-03-19

**Authors:** Clas Linnman, Lino Becerra, Alyssa Lebel, Charles Berde, P. Ellen Grant, David Borsook

**Affiliations:** 1 Pain and Analgesia Imaging Neuroscience (P.A.I.N.) Group, Department of Anesthesiology, Perioperative and Pain Medicine, Boston Children’s Hospital, Boston, Massachusetts, United States of America; 2 Center for Pain and the Brain, Harvard Medical School, Boston, Massachusetts, United States of America; 3 Center for Fetal Neonatal Neuroimaging and Developmental Science, Departments of Medicine and Radiology, Boston Children’s Hospital, Boston, Massachusetts, United States of America; City of Hope, United States of America

## Abstract

Evaluation of pain-induced changes in functional connectivity was performed in pediatric complex regional pain syndrome (CRPS) patients. High field functional magnetic resonance imaging was done in the symptomatic painful state and at follow up in the asymptomatic pain free/recovered state. Two types of connectivity alterations were defined: (1) Transient increases in functional connectivity that identified regions with increased cold-induced functional connectivity in the affected limb vs. unaffected limb in the CRPS state, but with normalized connectivity patterns in the recovered state; and (2) Persistent increases in functional connectivity that identified regions with increased cold-induced functional connectivity in the affected limb as compared to the unaffected limb that persisted also in the recovered state (recovered affected limb versus recovered unaffected limb). The data support the notion that even after symptomatic recovery, alterations in brain systems persist, particularly in amygdala and basal ganglia systems. Connectivity analysis may provide a measure of temporal normalization of different circuits/regions when evaluating therapeutic interventions for this condition. The results add emphasis to the importance of early recognition and management in improving outcome of pediatric CRPS.

## Introduction

Clinical features of post-traumatic complex regional pain syndrome (CRPS) include severe pain, hypersensitivity to noxious somatosensory stimuli (hyperalgesia), pain to non-noxious stimuli (allodynia), autonomic signs such as coldness, poor circulation, abnormal sweating, swelling and skin discoloration, motor abnormalities, including tremors and focal dystonias, and sometimes trophic signs such as abnormal hair and nail growth, muscle atrophy and joint contractures. CRPS is thought to involve peripheral and central sensitization of neuronal function [1], a view corroborated by recent neuroimaging studies [2], [3], [4], [5], [6], [7], [8], [9].

In children and adolescents, CRPS symptoms frequently fluctuate and often resolve within months to years [10], [11], a fortunate circumstance that offers an opportunity to study the CRPS brain longitudinally in the transition from symptomatic to asymptomatic. We have previously studied how functional *activation* in pediatric CPRS changes over the course of recovery [3]. In this follow up analysis on the same dataset, we address how pain changes the functional connectivity in the CRPS brain during the symptomatic state and whether such alterations also persist after symptom resolution. Functional connectivity analyses are based on temporal correlation in functional Magnetic Resonance Imaging (fMRI) Blood-Oxygen-Level-Dependent (BOLD) signal, allowing the testing of functional interactions between brain regions and how such interactions may be affected by experimental stimuli or diagnostic state.

We imaged pediatric patients with unilateral lower limb CRPS with painful cold stimulation of the affected and the unaffected limb on two occasions: while in the CRPS state and after symptom resolution. The laterality (affected/unaffected) by state (CRPS/resolved) within subject design allowed us to characterize pain networks longitudinally. We determined pain induced hyperconnectivity evoked by stimulating the affected limb in the CRPS state (as compared to the mirror unaffected limb) and also determined how such connectivity patterns may change with symptom resolution. We focused our analysis on pain induced functional connectivity of nine broad anatomical regions thought to be involved in the pathophysiology of CRPS: the amygdala (fear and anxiety), caudate, pallidum, putamen (motivational and movement related processes), thalamus (sensory processing), and the anterior cingulate-, insula- (sensory and affective components of pain as well as interoceptive processing), somatosensory- (pain location and intensity), and parietal-cortices (integrative processing and neglect).

## Methods

### Ethics statement

Written informed consent and patient assent were obtained from all subjects and their parents. The experimental procedure was approved by the McLean Hospital Institutional Review Board (for brain imaging) and the Children's Hospital Boston Institutional Review Board (for patient recruitment). Because this was a study involving pain in children, special procedures were adopted. One such safeguard was to halt the pain stimulus if the subjects reported a pain Visual Analog Score (VAS) of >8/10. In addition to parental consent, parents were present during all steps of the study. A post-scan evaluation questionnaire was completed by subjects to document their experience in the scanner and the painful stimuli they had received. In addition, as part of the IRB oversight, a report was sent to the IRB upon completion of each scanning session.

### Subjects

This dataset is identical to that presented in Lebel et al. [3] where we report pain and brush evoked functional *activations*. Briefly, eight pediatric CRPS patients aged 9–18 years (13.5±1.6 years, mean±SEM) were studied on two occasions — about 10 months apart — while in the CRPS state and after symptom recovery. Subjects with CRPS affecting the lower extremity unilaterally were recruited from the clinical caseload of the Chronic Pain Clinic at Children’s Hospital Boston. For functional magnetic resonance imaging during an attack, patients needed to have (i) refrained from using analgesic drugs at least 4 h prior to the examination; (ii) experienced a moderate to severe pain (i.e. pain intensity greater than 5 on a visual analog scale) and (iii) experienced unilateral limb pain. Exclusion criteria included (i) claustrophobia; (ii) significant medical problems such as uncontrolled asthma or seizure disorder, acute cardiac disease, psychiatric problems and other (non-CRPS) neurological disease; (iii) pregnancy; (iv) magnetic implants of any type and (v) weight >285 lbs.

### Experimental procedures

Prior to scanning, patients were tested in a quiet, temperature-regulated room at the Brain Imaging Center at McLean Hospital. Cold thresholds and responses to mechanical stimuli (pain intensity and defining the spatial extent of mechanical allodynia) were measured in the painful region within the ipsilateral-affected skin and in the corresponding contralateral (mirror) region. To determine cold pain thresholds, the skin was cooled down linearly at a slow rate (−1°C/s) until pain sensation was perceived, at which time the subject stopped the stimulus by pressing a button on a patient response unit (method of limits).

After completing the QST and the determining cold pain thresholds, subjects were placed in the magnet for functional imaging. After standard anatomical scans, functional scans were obtained in a semi-random sequence for brush and cold stimulation of the lower extremities. Two sets of four functional scans were collected for each side of the body, with two scans for brush (not used in this analysis) and two scans for 1°C below cold pain threshold on the affected side. For the cold scans, two pulses of cold stimuli (cold pain threshold −1°C; ramp: −4°C/s; duration 25 s stimulus interval: 30 s inter-stimulus interval) were applied to the same skin areas during both visits. Baseline temperature in each case was 32°C. Thermal stimuli were applied using a 3.0×3.0 cm^2^ Peltier thermode. These devices for use in the fMRI environment were developed at the Athinoula A. Martinos Center at the Massachusetts General Hospital with Medoc, Haifa, Israel. Subjects were scanned on a 3.0 T Trio (Siemens) using a quadrature Siemens head coil. Anatomical images were acquired using a magnetization prepared rapid gradient echo (MPRAGE) sequence. Functional resolution was 3.5×3.5×3.5 mm with a TR of 2.5 seconds.

Pain ratings (VAS 0–10) for the stimuli were obtained within the scanner using a turn-dial and visualized screen prompt. In addition, subjects were asked to complete a Post-Study Questionnaire following each study, see Lebel et al. [3] for further details.

### Rationale for ROI selection

Selecting regions for functional connectivity analyses can either be done by a separate functional localization scan not used for connectivity analyses, by *a priori* anatomical regions of interest (ROI) definitions, or by identifying task positive regions in the general linear model and applying those clusters to follow-up connectivity analyses on the same data (circular analysis [12]). Here, we chose the *a priori* anatomical approach, averaging all voxels in a pre-defined region of interest. Prior publications from adult CRPS imaging studies provide a rationale for our ROI selection as follows: *Amygdala*: reduced opioid binding potential [13]; *Caudate*: activation to pain and reduction after treatment [2], [14]; *Pallidum*: white matter tract alterations [9]; *Putamen*: decreased responses after treatment [2]; *Thalamus*: elevated resting perfusion in subacute CRPS [15], [16], increased metabolism [17], decreased blood flow pre-treatment [18], increased blood flow after spinal cord stimulation in a mixed cohort [19]; *Anterior cingulate*: hyperactive in contrast to unaffected limb stimulation [8], altered white matter tracts [9], less activation during pain suppression in CRPS [20], decreased activation with treatment [21]; *Insula*: hyperactive in contrast to unaffected limb stimulation [8], more active to pain in CRPS [14], opioid receptor binding negatively correlated with pain [13]; *Somatosensory cortex*: hyperactive in contrast to unaffected limb stimulation [8], decreased activation correlated with pain relief [21], altered delta and theta range activity [22]; *Parietal cortex*: hyperactive in contrast to unaffected limb stimulation [8], increased blood flow after spinal cord stimulation in a mixed cohort [19].

### Brain flipping and data preprocessing

Preprocessing of functional data was done in SPM8 with slice timing correction, realignment and co-registration to structural MPRAGE images and normalization to the MNI 152 template with parameters derived from the structural data. For subjects that had their right leg affected (two out of the eight subjects), brains were flipped along the y-axis (anterior–posterior axis) as we have previously described [3], [23] to allow for inter-subject comparisons. In order to contrast intra-subject functional connectivity in the affected and unaffected sides, each subject’s functional time series data from the unaffected side stimulation was flipped along the y-axis (anterior–posterior axis) before being registered to the standard brain. In other words, both right (unaffected and flipped data) and left (affected) sided stimulation should lead to right sided somatosensory cortex activation. Thus, the analysis made an assumption of hemispheric symmetry of pain processing in order to allow for a within subject contrast of affected versus unaffected limb stimulation.

After preprocessing, data was fitted to a first level model that included boxcar functions for cold ramp-up, cold stimulation, cold ramp-down and six motion parameters derived from the realignment procedure. General linear model results have been reported previously [3].

### Psychophysiological interaction analyses

Psycho-physiological interaction (PPI) analysis tests how much of the variance of BOLD signal can be explained by the interaction between signal in one “seed” region of interest (the physiological parameter) and an experimental variable (pain) [24].

In the present case, the PPI indicates regions that are more functionally connected to the seed region only during pain stimulation. The model also included the seed region time series and the task. This way, any signal that is better explained by the seed or by the task will fall into those residuals, and the remaining PPI term describes unique stimulus driven functional connectivity.

The nine anatomically defined seed regions—amygdala, caudate, pallidum, putamen and the thalamus, and anterior cingulate-, insula-, somatosensory-, and parietal-cortices—was defined in the contralateral (right) hemisphere according to the AAL-atlas [25], see Figure S1 and Table S1 for details, ROIs are available at (http://www.cyceron.fr/web/aal__anatomical_automatic_labeling.html).

The seed region average voxel time series was extracted, hemodynamically deconvolved [26] and element-by element multiplied with the experimental parameter (cold stimulation) resulting in the PPI interaction term. The first level PPI design matrix included the interaction term, the psychological parameter, and the seed time-series. The six motion correction parameters were also included into the model to further account for possible movement induced artifacts. The interaction term identifies voxels in the brain that display a difference in regression slope dependent on the seed time-series and the experimental condition. The fit of this model is mapped into an SPM image for each participant, technically equivalent to a first level univariate analysis. For each subject, PPI effects were estimated at each voxel, and contrast maps were produced.

### Second level PPI analysis

**Figure 1:**
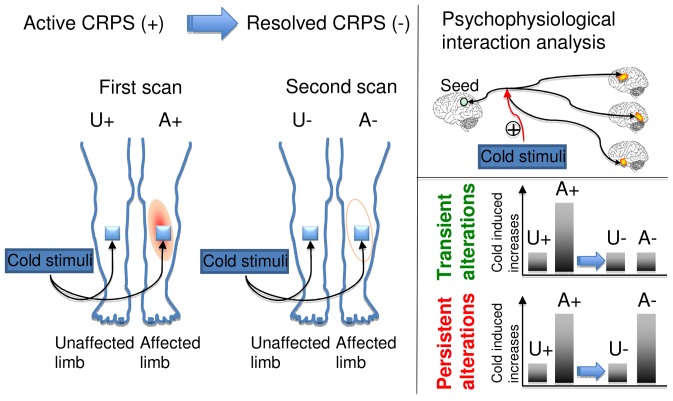
Overview of study procedure. Patients were scanned on two occations, in the symptomatic CRPS state (+) and after symptom resolution. On both occations, cold stimuli was applied to the painful region in the affected limb and to the corresponding unaffected limb. The analysis focused on pain-induced changes in functional connectivity (psychophysiological interaction, PPI) that were greater when stimulating of the affected limb in the CRPS+ state as compared to the unaffected limb in the CPPS+ state. As an additional criteria, the cold induced changes were either characterized as transient, i.e. no difference in the CRPS- state, or persistent, i.e. also greater in the CRPS- state.

Individual PPI contrast images were entered into a second-level repeated measurements analysis using a factorial design including three factors (subject, affected/unaffected, and CPRS/resolved). The resulting ANOVA model allowed for contrasting cold-induced connectivity changes in the affected versus unaffected limb, and in the CRPS versus the recovered state (see Figure 1).

The analysis focused on two contrasts: First, we identified regions that show increased cold-induced functional connectivity in a) the affected limb, b) the unaffected limb in the CRPS state, c) the (previously) affected limb in the recovered state, and d) the (always) unaffected limb in the recovered state. To identify regions that displayed hyperconnectivity only during stimulation of the affected limb in the CRPS state, we contrasted a>b, but c∼d. We refer to this contrast as *transient alterations*. Second, we identified regions that displayed hyperconnectivity during stimulation of the affected limb in the CRPS state, and that also displayed hyperconnectivity despite recovery by contrasting a>b and c>d. This contrast is referred to as *persistent alterations* (See Figure 1 for an illustration). Clusters exceeding the family wise error correction criteria p<0.05 were considered significant. Anatomical labeling was done by visual inspection and confirmed by automated labeling through the Talairach [27] demon (www.talariach.org) after transformation of the MNI coordinates using the tal2mni algorithm (http://imaging.mrc-cbu.cam.ac.uk/downloads/MNI2tal/mni2tal.m).

## Results

### Pain ratings

As detailed previously [3], average spontaneous pain rating prior to the first scan was 5.1±1.6 (mean±SEM) on a VAS scale of 0–10 and no spontaneous pain at the time of the second scan. The average cold pain threshold in the CRPS+ state was 5.9±0.2°C, and 2.1±0.3°C in the recovered state (p<0.01).

During scanning, pain rating for cold stimulation was significantly higher for the affected (VAS = 5.2±0.4) versus unaffected (VAS = 0.8±0.1) limb in the CRPS state (p<0.01). In the recovered state, pain ratings were dramatically lower then in the CRPS state (p<0.01), but the pain ratings for cold stimulation of the affected limb (VAS = 2.3±0.5) was still higher than for the unaffected limb (VAS = 1.2±0.5), p<0.01.

### Functional connectivity results

**Figure 2:**
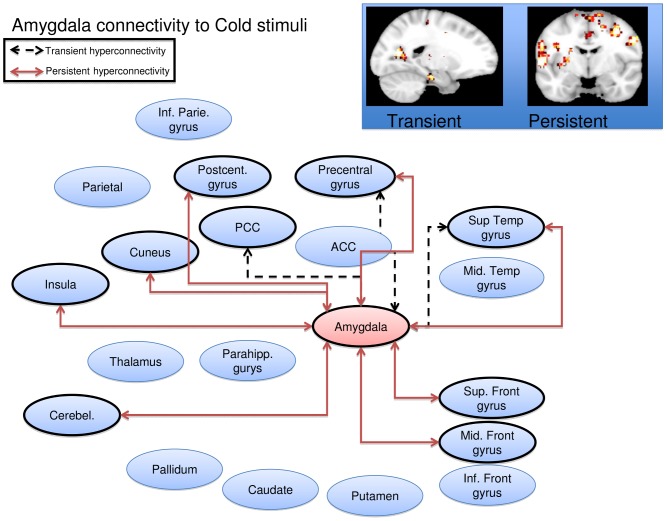
Amygdala seed wire diagram of regions that displayed significant (p_fwe_<0.05) altered connectivity patterns. Transient alterations are indcated with a dashed line, persistent alterations are indicated with a solid red line. Brain maps are displayed at a sagital section through x = −18 for transient alerations, and a coronal section through y = −6 for persistent alterations. Maps are thresholded at 3<T<4. See Table 1 for coordinates and statistics.

**Figure 3:**
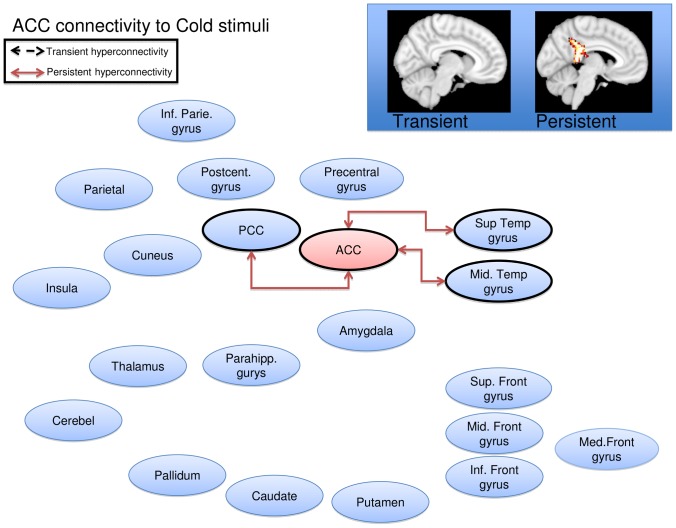
Anterior cingulate seed wire diagram of regions that displayed significant (p_fwe_<0.05) altered connectivity patterns. No transient alterations were found, persistent alterations are indicated with a solid red line. Brain maps are displayd at a sagital section through x = 0 for (no) transient alerations, and x = −5 for persistent alterations. Maps are thresholded at 3<T<4. See Table 1 for coordinates and statistics.

**Figure 4:**
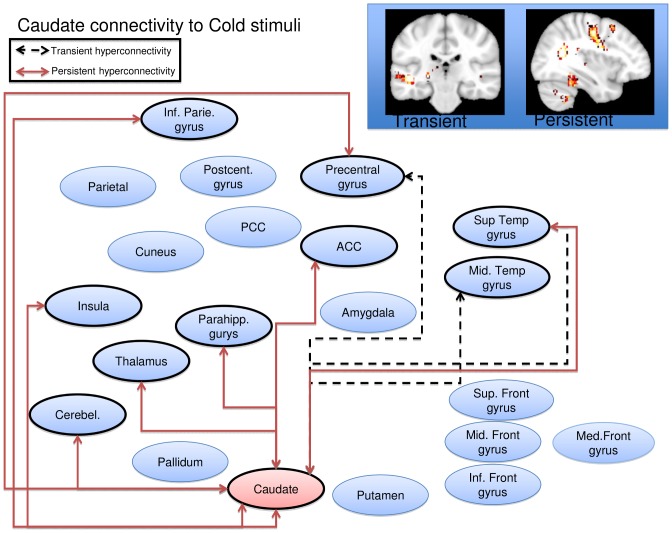
Caudate seed wire diagram of regions that displayed significant (p_fwe_<0.05) altered connectivity patterns. Transient alterations are indcated with a dashed line, persistent alterations are indicated with a solid red line. Brain maps are displayed at a coronal section through y = −26 for transient alerations, and a sagital section through x = 36 for persistent alterations. Maps are thresholded at 3<T<4. See Table 1 for coordinates and statistics.

**Figure 5:**
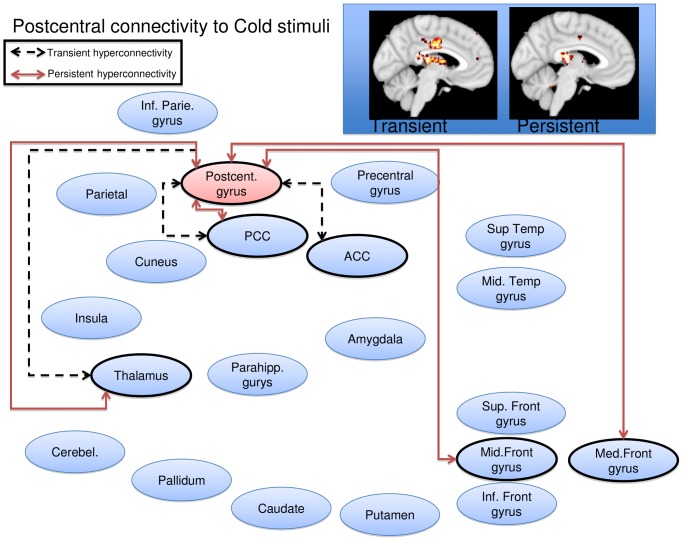
Postcentral gyrus seed wire diagram of regions that displayed significant (p_fwe_<0.05) altered connectivity patterns. Transient alterations are indcated with a dashed line, persistent alterations are indicated with a solid red line. Brain maps are displayd at a sagital section through x = −4 for transient alerations, and x = −4 for persistent alterations. Maps are thresholded at 3<T<4. See Table 1 for coordinates and statistics.

**Figure 6:**
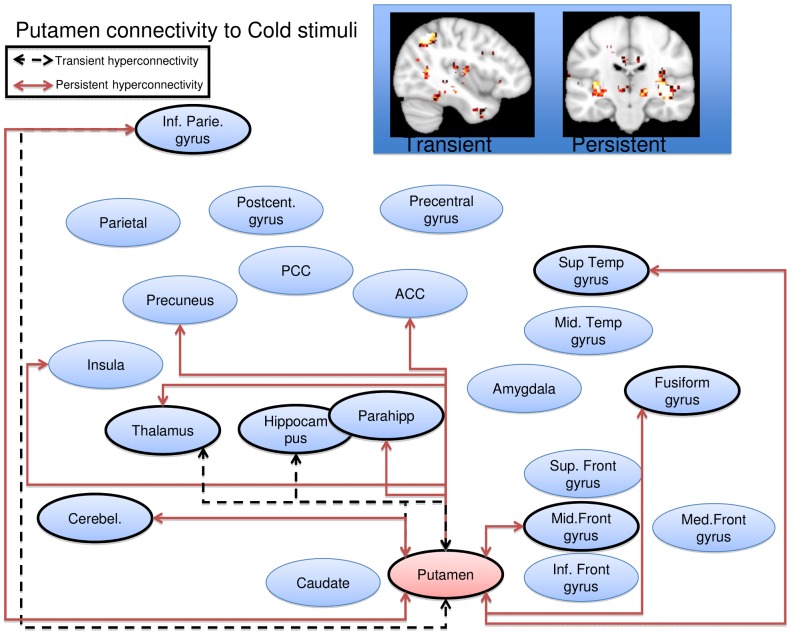
Putamen seed wire diagram of regions that displayed significant (p_fwe_<0.05) altered connectivity patterns. Transient alterations are indcated with a dashed line, persistent alterations are indicated with a solid red line. Brain maps are displayd at a sagital section through x = −40 for transient alerations, and a coronal section through y = −20 for persistent alterations. Maps are thresholded at 3<T<4. See Table 1 for coordinates and statistics.

Cold stimulation of the affected CRPS limb in the symptomatic state led to a general pattern of increased functional connectivity between the seed regions and the brain, consistent with pain leading to an increased degree of BOLD synchronization within pain processing regions. Some, but not all, of the elevations were present also in the symptomatically recovered state, as specified below. Notably, there were no regions displaying transient or persistent cold induced *reductions* in functional connectivity. Of the nine included seed regions evaluated (amygdala, anterior cingulate, caudate, insula, pallidum, parietal cortex, postcentral gyrus, putamen, and thalamus), five displayed significant (Family Wise Error corrected p<0.05) elevations in functional connectivity during stimulation of the affected limb as compared to stimulation of the unaffected limb during the symptomatic and/or recovered CRPS state. Cartoon wire diagrams and brain maps (Figures 2, 3, 4, 5, 6), along with Table 1 specifying coordinates and significance of alterations, are provided in an effort to provide an overview of the observed alterations. Several broad anatomical regions are indexed as having both transient and persistent alterations. The localization of peaks of such alterations are indicated in Table 1.

**Table 1:** Significant psychophysiological interaction effects.

Seed	Cluster	Cluster	Peak	MNI	Peak region
	p(FWE-corr)	size	Z	x y z	
**Amygdala**					
**Transient alterations**					
	0.006	223	3.19	42−5022	Right Superior Temporal Gyrus
					
	0.005	232	4.67	−20−6812	Left Posterior Cingulate
			3.71	−18−58 8	Left Posterior Cingulate
			3.66	−12−7010	Left Cuneus
					
	0.000	373	4.52	−56−88	Left Superior Temporal Gyrus
			4.48	−50−12−8	Left Superior Temporal Gyrus
			4.15	−56−120	Left Superior Temporal Gyrus
					
	0.000	382	4.19	54−434	Right Precentral Gyrus
			4.04	38−1432	Right Precentral Gyrus
			3.95	46−1230	Right Precentral Gyrus
					
	0.045	139	4.06	−36−1646	Left Precentral Gyrus
			3.35	−44−2452	Left Postcentral Gyrus
					
	0.030	155	4.05	58−4614	Right Superior Temporal Gyrus
			3.45	60−5226	Right Supramarginal Gyrus
			3.41	62−4020	Right Superior Temporal Gyrus
					
					
					
**Persistent alterations**					
	0.021	170	5.33	−323022	White matter
			3.55	−342214	Left Insula
			3.22	−263418	White matter
					
	0.001	332	4.72	−60−66	Left Superior Temporal Gyrus
			4.55	−58−228	Left Precentral Gyrus
			3.7	−54−616	Left Precentral Gyrus
					
	0.000	1004	4.7	58−836	Right Precentral Gyrus
			4.57	36−646	Right Middle Frontal Gyrus
			4.54	48−1630	Right Precentral Gyrus
					
	0.000	420	4.46	−46−3212	Left Superior Temporal Gyrus
			4.1	−60−3618	Left Superior Temporal Gyrus
			3.84	−54−3412	Left Superior Temporal Gyrus
					
	0.000	1359	4.34	−16−4860	Left Precuneus
			4.33	−16−3864	Left Postcentral Gyrus
			4.09	14−4854	Right Precuneus
					
	0.026	161	4.33	22−38−32	Right Cerebellum, Anterior Lobe
			3.56	14−44−26	Right Cerebellum, Culmen
			3.32	20−48−30	Right Cerebellum, Anterior Lobe
					
	0.011	196	4.21	41468	Right Superior Frontal Gyrus
			3.75	10−270	Right Superior Frontal Gyrus
			3.58	4−464	Right Medial Frontal Gyrus
					
	0.027	159	4.16	−40−1848	Left Precentral Gyrus
			3.18	−30−1658	Left Precentral Gyrus
					
	0.009	204	3.8	54−548	Right Superior Temporal Gyrus
			3.79	60−3820	Right Insula
			3.71	58−4614	Right Superior Temporal Gyrus
					
					
**Anterior cingulate**					
**Persistent Alterations**					
	0.000	737	4.51	−6−4018	Left Posterior Cingulate
			4.49	−6−428	Left Posterior Cingulate
			4.15	−10−4232	Left Cingulate Gyrus
					
	0.026	140	3.89	60−544	Right Middle Temporal Gyrus
			3.84	40−5626	Right Superior Temporal Gyrus
			3.69	46−5612	Right Superior Temporal Gyrus
					
**Caudate**					
**Transient alterations**					
	0.042	150	4.6	−44−26−4	Left Superior Temporal Gyrus
			3.47	−58−28−4	Left Middle Temporal Gyrus
					
	0.001	346	4.44	30−5424	White matter
			4.26	46−5412	Right
			3.93	58−5010	Right Superior Temporal Gyrus
					
	0.037	155	4.4	−50−466	Left Middle Temporal Gyrus
			3.59	−56−4216	Left Superior Temporal Gyrus
					
	0.023	176	4.28	40−832	Right Precentral Gyrus
			4.24	36030	Right Precentral Gyrus
					
**Persistent alterations**					
	0.000	492	5.45	30−5424	No Gray Matter found
			5.16	36−5616	Right Superior Temporal Gyrus
			4.27	26−3826	Right Insula
					
	0.000	1471	5.05	−26−3610	Left Caudate
			4.58	−22−260	Left Thalamus
			4.41	−42−24−8	Left Suprior Temporal Gyrus
					
	0.000	377	4.75	−38−6244	Left Inferior Parietal Lobule
			4.31	−44−5642	Left Inferior Parietal Lobule
			3.9	−42−5634	Left Inferior Parietal Lobule
					
	0.000	418	4.28	−16−2654	Left Precentral Gyrus
			4.13	−16830	Left Cingulate Gyrus
			3.98	−18−2840	Left Cingulate Gyrus
					
	0.000	447	3.98	20242	Right Cingulate Gyrus
			3.98	34−834	Right Precentral Gyrus
			3.96	20−432	Right Caudate
					
	0.049	144	3.79	42−44−24	Right Cerebellum, Culmen
			3.67	34−40−22	Right Fusiform Gyrus
			3.54	40−36−12	Right Parahippocampal Gyrus
**Postcentral gyrus**					
**Transient alterations**					
	0.000	280	4.14	−8−2812	Left Thalamus
			3.75	10−4018	Right Posterior Cingulate
			3.69	−4−2012	Left Thalamus
					
	0.001	260	4.11	2−1234	Right Cingulate Gyrus
			3.74	−4−2032	Left Cingulate Gyrus
			3.41	6−2036	Right Cingulate Gyrus
					
**Persistent alterations**					
	0.006	182	4.4	−4−2612	Left Thalamus
			3.76	−8−3818	Left Posterior Cingulate
			3.61	−18−288	Left Thalamus
					
	0.030	129	4.18	14−1260	Right Medial Frontal Gyrus
			4.06	24−1662	Right Middle Frontal Gyrus
**Putamen**					
**Transient alterations**					
	0.006	214	4.73	−40−6048	Left Inferior Parietal Lobule
			3.84	−44−5638	Left Inferior Parietal Lobule
			3.78	−40−5052	Left Inferior Parietal Lobule
					
	0.016	173	4.43	−22−262	Left Thalamus
			4.32	−12−268	Left Thalamus
			3.85	−26−24−6	Left hippocampus
**Persistent alterations**					
	0.004	229	4.96	38−202	Right Claustrum
			4.64	42−22−6	Right Insula
			3.43	34−2010	Right Claustrum
					
	0.001	291	4.82	32−842	Right Middle Frontal Gyrus
			3.89	36−852	Right Middle Frontal Gyrus
			3.85	14246	Right Cingulate Gyrus
					
	0.000	489	4.61	−24 −300	Left Thalamus
			4.15	−12−264	Left Thalamus
			4.07	−32−24−4	Left Lentiform Nucleus
					
	0.002	252	4.47	−32−32−26	Left Parahippocampal Gyrus
			4.18	−36−46−20	Left Fusiform Gyrus
			3.67	−32−38−16	Left Fusiform Gyrus
					
	0.000	685	4.35	−42−5444	Left Inferior Parietal Lobule
			4.3	−44−5636	Left Inferior Parietal Lobule
			4.02	−42−5030	Left Supramarginal Gyrus
					
	0.026	154	4.35	60−1214	Right Transverse Temporal Gyrus
			3.42	56−104	Right Superior Temporal Gyrus
					
	0.018	168	4.23	−12−56−32	Left Cerebellum, Anterior Lobe
			3.37	−10−64−22	Left Cerebellum, Declive
			3.35	−6−60−42	Left Cerebellum, Uvula
					
	0.000	620	4.08	−14−4046	Left Paracentral Lobule
			4.05	−16−4852	Left Precuneus
			3.87	−20−3438	Left Cingulate Gyrus

Transient alterations were defined as a cold-induced increase in functional coupling between seed region and clusters present when contrasting cold stimulation to the affected limb versus in the CRPS state versus a) the unaffected limb in the CRPS state; b) the affected limb in the recovered state; and c) the unaffected limb in the recovered state. Persistent alterations were defined as regions with a cold-induced increase in functional coupling between seed region present in both the CRPS state (versus unaffected limb) and in the symptomatically recovered state (affected vs unaffected limb). Cluster size for entire clusters, with sub-peak coordinates. Regions indicate nearest (<5 mm) gray matter.

#### Amygdala

Transient increased connectivity (defined as greater connectivity changes from stimulating the affected limb in the symptomatic state as compared to the unaffected limb and as compared to the recovered state) was observed to the superior temporal gyrus, the posterior cingulate, cuneus, precentral gyrus and the supramarginal gyrus. Persistently increased connectivity (defined as greater connectivity changes from stimulating the affected limb in both the symptomatic and recovered state versus the unaffected limb) were observed to the insula, superior temporal gyrus, precentral gyrus, middle frontal gyrus, precuneus, postcentral gyrus, the culmen of the cerebellum, superior frontal gyrus and the medial frontal gyrus, see Figure 2.

There were no regions displaying transient or persistent cold induced *reductions* in functional connectivity.

#### Anterior Cingulate

Transient increased connectivity (defined above) was observed to the posterior cingulate gyrus, and to the middle and superior temporal gyrus (Figure 3). There were no regions displaying persistent increases, or transient or persistent cold induced *reductions* in functional connectivity.

#### Caudate

Transient increased connectivity was observed to the middle and superior temporal gyrus. Persistent increased connectivity was observed to the superior temporal gyrus, insula, within the caudate, to the thalamus, the inferior parietal lobule, the precentral gyrus, the cingulate gyrus, the culmen of the cerebellum, the fusiform gyrus and the parahippocampal gyrus (Figure 4). There were no regions displaying transient or persistent cold induced *reductions* in functional connectivity.

#### Postcentral gyrus

Transient increased connectivity was observed to the thalamus and to the posterior and middle cingulate gyrus. Persistent increased connectivity was observed to the thalamus, posterior cingulate and to the medial frontal gyrus (Figure 5). There were no regions displaying transient or persistent cold induced *reductions* in functional connectivity.

#### Putamen

Transient increased connectivity was observed to the inferior parietal lobule, the thalamus and to the hippocampus. Persistent increased connectivity were observed to the claustrum, insula, middle frontal gyrus, cingulate gyrus, thalamus, putamen, parahippocampal gyrus, fusiform gyrus, inferior parietal lobule, supramarginal gyrus, transverse and superior temporal gyrus, the cerebellar dentate, the ulvula, paracentral lobule and the precuneus (Figure 6). There were no regions displaying transient or persistent cold induced *reductions* in functional connectivity.

## Discussion

We demonstrate stimulus-induced increases in functional connectivity in pediatric CRPS. These increases include ones that diminish after symptomatic recovery and others that appear to persist despite return of normal limb function and normalized pain. These results may indicate both transient and persistent changes, the length of which we have not yet determined. Changes may be mediated by mechanisms such as a cortical reorganization in response to trauma and CRPS development that leaves its mark on the brain’s connectivity patterns at remission. In general, we did not observe any transient or persistent *reductions* in functional connectivity, consistent with the observation that pain stimulation of an affected or unaffected limb leads to afferent nociceptive inflow that engages multiple structures involved in pain processing, thereby increasing their functional coupling [28].

The postcentral gyrus, containing the primary somatosensory cortex, displayed both transient and persistently altered functional connectivity to the thalamus. This may suggest that CRPS patients not only show an elevated processing of nociceptive inflow from the affected limb via the thalamus to S1, but also that this elevation persists after resolution of pain. One speculation is that the prolonged peripheral nociceptive inflow in the CRPS state leads to Hebbian learning that elevates connectivity in the primary nociceptive circuit. This may result in a connective pattern that persists despite symptom resolution. The imbalance between transient changes (perhaps getting weaker over time) and persistent alterations (lasting beyond symptom resolution) may provide an insight into the adaptive processes (plasticity) involved in healing. As pediatric CRPS may relapse after an additional trauma [29], [30], the observed persistent alterations may constitute a risk factor. How long such an elevation remains, and if such elevations constitute a risk factor for pain syndromes later in life, remains to be explored. Chronic adult CRPS brain appear to affect regions involved in emotional behaviors such as the hippocampus [31] and ventromedial prefrontal cortex [9], and also in motor circuits [4]—possibly being secondary and (mal-)adaptive to the persistent toll of unrelenting pain. Clearly, this underscores the need for an early diagnosis and treatment.

Of the nine seed regions evaluated, two cortical regions—the anterior cingulate cortex (ACC) and postcentral gyrus (PCG)—and three subcortical regions—the amygdala, caudate and putamen—showed significant alterations.

### Cortical Regions

A number of cortical regions show changes in adult and pediatric CRPS [3], [4], [7], [8], [9], particularly the ACC and PCG. The ACC is involved in a wide range of behavior including pain processing and cognitive/emotional regulation [32]. With the ACC as a seed region, we observed persistent alterations (i.e., a significantly higher stimulus induced shift in connectivity in the affected limb both in the symptomatic and in the recovered state) within the regions of the posterior cingulate and temporal lobe. The temporal lobes connectivity patterns have been considered to play a role in the uncertainty of decision-making [33]. Functional connectivity appears to be higher between the ACC and temporal regions when uncertainty is higher, which may correspond, in the present context, to persistent fear of pain after symptom recovery. We did not, however, collect measures of pain fear and kinesophobia to confirm this speculation.

Notably, when using the caudate and putamen seed, persistent hyperconnectivity was observed to the mid- and posterior-cingulate respectively. Models of cingulate function in pain associate anterior mid cingulate regions with emotional processing, whereas mid and posterior regions are more involved in skeletomotor orientation [34].

Changes observed in the PCG showed the two types of hyperconnectivity: (1) transient to the ACC and PCC and (2) persistent to the thalamus and frontal gyri. The PCG is a main region for interpreting sensory information. The transient connectivity changes may reflect processes that include information relating to the interpretation of a stimulus and the subsequent sequencing of its salience (cingulate gyrus) [35], [36]. The more persistent changes seem related to the ability of sensory stimuli to drive inputs related to cognitive processing. It is well known that cognition may be altered in CRPS [37], [38] and this circuit may be altered even with symptomatic recovery. Such changes would clearly have implications for determining “back to normal” activities in children.

### Subcortical Regions

Changes in the amygdala and basal ganglia were observed. In the case of the amygdala, the middle and superior frontal gyri were two of the structures to which the amygdala displayed persistently altered functional connectivity.

These patients have, in their symptomatic condition, continuous ongoing pain and potential for evoked stimuli (clothes, bumping into objects etc.). One interpretation of this is that the amygdala is involved in fear conditioning [39], with cognitive interpretation of this behavior mediated through the observed persistent functional connections. Moreover, decreased opioid receptor binding potential has been observed in the amygdala in adult CRPS [13]. Notably, some cognitive treatments may contribute to limiting fear conditioning through diminishing this hyperconnectivity between these structures [40]. Persistent hyperconnectivity of the amygdala was also observed in other regions, including the cerebellum (known to be involved in pain and aversive processing and sequencing of information [41]) and the postcentral gyrus and precuneus. Notably, a recent study found the amygdala-to-precuneus functional connectivity to be highly relevant in subliminal fear conditioning [42]. In line with this, exposure (extinction) based therapies may be successful in CRPS [43].

The basal ganglia play an important role in pain processing [44], [45]. In this study we observed significant hyperconnectivity changes in the caudate and in the putamen. In the case of the caudate transient hyperconnectivity was seen only with cortical regions (temporal regions, the PCG) while persistent hyperconnecitvity was observed with cortical (inferior parietal, PCG, insula, superior temporal gyrus, parahippocampal gyrus) and subcortical regions (thalamus and cerebellum). The caudate has been implicated in chronic pain conditions including migraine [46] and fibromyalgia treatment responses[47] to mention a few. The role of the caudate in pain is unknown, but may be part of integrated processes (sensory, cognitive, emotional, motoric) that include processes involved in conscious or treated pain suppression [48], [49].

In the case of the putamen, transient hyperconnectivity was seen with the hippocampus and thalamus and persistent hyperconnectivity included multiple regions (cerebellum, insula, parietal lobe, precuneus, ACC, fusiform gyri and temporal lobe). Based on studies in patients with putaminal brain lesions, the structure has been considered to “*contribute importantly to the shaping of an individual subjective sensory experience by utilizing internal cognitive information to influence activity of large areas of the cerebral cortex*” [50]. In addition, measures of the effects of analgesics, including opioids [51] and anesthetics [52], show that the region is reportedly significantly activated, suggesting a potential role in analgesia (with normal function).

Taken together, the involvement of basal ganglia in persistent pain is further supported in these studies. These regions receive inputs from all cortical areas and, throughout the thalamus, project principally to frontal lobe areas thus having multiple roles including shifting attention, motor planning, reinforcing wanted behavior and suppressing unwanted behavior [53]. All these have clinical correlates in the CRPS pain behavioral phenotype. Behavioral correlates of altered basal ganglia hyperconnectivity were not specifically measured. However, in caudate lesioned animals, there is altered contralateral paw contact placing reaction and paw usage [54]. The activation analysis in our prior study on this dataset found activation in the basal ganglia evoked by cold and brush stimuli[3]. Thus, in CRPS, the interaction with the environment may be compromised in part by alterations of caudate and other basal ganglia function, including approach/avoidance reactions [55].

### Relationship to functional activation studies and resting state connectivity

Functional connectivity and functional activation describe two different aspects of the brain. Regions that are activated by an experimental stimulus (i.e. cold stimulation of a limb) do not necessarily display signal coherence or functional connectivity. That said, there are some notable similarities between the present results and the results from the univariate analysis published previously [3]. In that study (Lebel et al., 2008), we observed that functional alterations (activation to cold or brush) persisted even after complete resolution of pain symptoms and that activations were observed in all the areas evaluated here. These findings suggest that both the corresponding regions and connectivity patterns are significantly altered.

Several recent studies have demonstrated alterations in resting state functional connectivity in adults with chronic clinical pain [56], [57], [58], [59], indicating that connectivity patterns can be altered either without symptom provocation or with spontaneous fluctuating clinical pain.

### Limitations

There are some caveats to this study that need to be acknowledged.

- Sample size: Eight subjects is a small sample size, despite being a within subject design. Initially we enrolled 12 patients, but technical issues, excessive motion (a particular problem in pediatric populations), and subject dropout following resolution of CRPS limited the number of complete datasets. Despite the stringent statistical corrections thresholds used, the results need to be replicated in a larger sample.
- Interscan times: Another potential limitation is the time between the initial scan and the resolution of CRPS. The average time between the first two scans was 303 days, potentially leading to morphological and functional changes of normal brain maturation that are unrelated to the resolution of CRPS. Future studies might include an age matched healthy control group that is scanned on two equally spaced occasions.
- Asymmetric brain changes: In the CRPS brain, there is evidence for significant interhemispheric asymmetry between the motor cortical representation of affected and unaffected limbs [60]. This is an asymmetry we sought to capitalize on by the right-left flipping procedure, thereby analyzing somatosensory activations from the affected and unaffected limb in the stereotactic space. This allowed us to directly compare stimulus induced connectivity changes between the hemispheres. The downsides of this approach are that cortical organization for the left and right hemisphere may not be sufficiently symmetric to equate the respective hemisphere, and that some functional pain processing pathways may not be symmetrically distributed in the healthy brain. As our sample size was limited, an analysis of potential disease by laterality interactions was not feasible.
- Seed Regions: We used large seed regions defined from atlas space rather than functionally defined ROIs for two reasons: (1) to avoid ‘double dipping’ as we did not have a separate functional localization task, and (2) to achieve a more stable region time course estimates by including multiple voxels in large ROIs. A downside of this is that seeds will contain both signal related to the task and non-related signal or noise. As such, we are somewhat reducing the chances of identifying significant, stimulus driven connectivity changes; i.e. a higher risk for Type II errors. It is conceivable, for example, that by dividing the amygdala into subnuclei, we would have observed differential relationships of the central and basolateral nuclei [61], [62]. However, having many small seeds create a greater multiple comparison problem.
- Head motion: Subject head motion during scanning leads to lower SNR and influences measures of functional connectivity [63]. In the current study, the mean motion (the Euclidian distance displacement of each brain volume as compared to the previous volume) was 0.089 mm. This is in the upper range of what has been observed in resting state studies in a large young adult sample (n = 1000, aged 18 to 30 yrs., mean motion = 0.05 mm) [63], but is below the mean motion observed in resting state scans of a large youth sample (n = 456 aged 8–23 yrs., mean motion = 0.14 mm) [64]. We attempted to remedy for potential effects of subject motion in two ways. First, the motion parameters from data preprocessing were included as nuisance variables in all models. Second, subject mean motion was calculated and compared with no significant differences within subjects across stimulation of the affected versus unaffected limb, across the CRPS positive versus CRPS resolved state, and no limb ⊥ state interaction.

## Conclusions

These results support our prior observations that among pediatric CRPS patients, alterations in brain systems persist even after functional recovery and marked reduction in pain intensity. The significance of these changes may be reflected in symptoms or behaviors that are known to be modulated by some of the structures evaluated in this study. The development of persistent alterations in functional connectivity is especially concerning because complete remission may take longer than overt symptom resolution. There may be an opportunity to use such measures to determine health trajectories and intervention effectiveness. If hyperconnectivity patterns persist, this may set the stage for later reoccurrence, as is frequently observed after re-injury [29], [30].

Present findings, indicative of pain induced persistent reorganization of the cortical, limbic and basal ganglia circuits, add emphasis to the importance of early recognition and management as a major factor in improving outcome and preventing resistant CRPS [30].

## Supporting Information

Figure S1 **ROI definitions from the Automated Anatomical Labeling (AAL) library.**
(TIFF)

Table S1 **ROI sizes and centers of gravity from the the Automated Anatomical Labeling (AAL) library.**
(DOC)
