# Supplementary figures and images for: Transient and Persistent Pain Induced Connectivity Alterations in Pediatric Complex Regional Pain Syndrome
Source: PLoS One. 2013 Mar 19;8(3):e57205. doi: 10.1371/journal.pone.0057205 (PMC3602432; doi:10.1371/journal.pone.0057205)

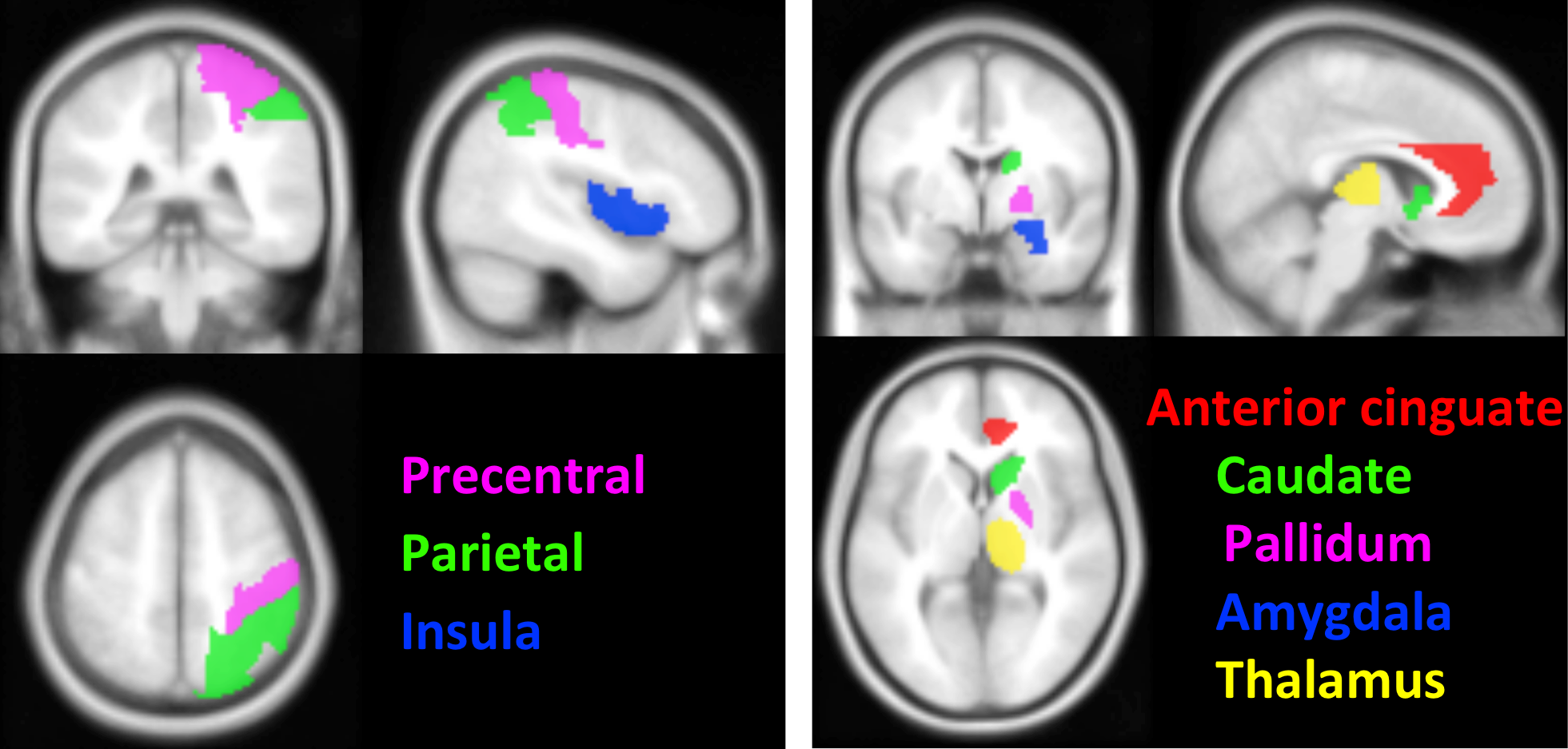

Supplement: Figure S1 — ROI definitions from the Automated Anatomical Labeling (AAL) library. (TIFF) [file pone.0057205.s001.tiff]
