# Supplementary material for: Transient and Persistent Pain Induced Connectivity Alterations in Pediatric Complex Regional Pain Syndrome
Source: PLoS One. 2013 Mar 19;8(3):e57205. doi: 10.1371/journal.pone.0057205 (PMC3602432; doi:10.1371/journal.pone.0057205)
Supplement: Table S1 — ROI sizes and centers of gravity from the the Automated Anatomical Labeling (AAL) library. (DOC) [file pone.0057205.s002.doc]

Table S1

ROI name MNI Center of Gravity Volume

X Y Z (mm^3^)

R. Anterior cingulate 8.1 35.7 14.4 10504

R. Amygdala 27.1 -0.6 -18.8 1984

R. Caudate 14.5 10.8 8.1 7952

R. Insula 38.7 5.0 0.8 14160

R. Pallidum 20.9 -1.1 -1.1 2240

R. Parietal lobe 33.6 55.6 56.0 28536

R. Postcentral gyrus 41.2 -26.8 51.3, 30584

R. Putamen 27.5 3.7 1.2 8512

R. Thalamus 12.7 -18.7 6.7 8456
